# Opioid Prescribing Behavior in Emergency Settings and Associated Risk Factors in the Central Region of the Kingdom of Saudi Arabia

**DOI:** 10.7759/cureus.98317

**Published:** 2025-12-02

**Authors:** Khalid A Ateyyah, Ahmed H Almutairi, Rammaz H Khoja, Rahaf H Khoja, Abdullah A Najjar, Releman H Alsharif, Yazeed M Asiri, Ali A Al Sarrar

**Affiliations:** 1 Medicine/Emergency Medicine, Taibah University, Madinah, SAU; 2 Emergency Medicine, Majmaah University, Al Majma'ah, SAU; 3 Medicine, Taibah University, Madinah, SAU; 4 Emergency Medicine, King Fahad Hospital, Madinah, SAU; 5 Emergency Medicine, Ministry of National Guard-Health Affairs, Jeddah, SAU; 6 Medicine, College of Medicine, Taibah University, Madinah, SAU

**Keywords:** emergency physicians, healthcare practice, opioid prescribing, pain management, prescribing behavior, saudi arabia

## Abstract

Background: Pain management is an important issue in emergency medicine. Although opioids have many associated adverse effects, including opioid misuse and dependence, they are still frequently prescribed nowadays. Subsequently, it is vital to understand the factors influencing opioid prescribing decisions among emergency physicians. This article aims to examine factors affecting opioid prescribing behaviors among emergency physicians in Saudi Arabia and assess attitudes impacting prescribing practices.

Methods: A cross-sectional study was performed in the period from February 2024 to February 2025 among 213 emergency physicians in Saudi Arabia. The clinical, external, and psychosocial factors affecting opioid prescribing decisions were evaluated using a 22-item Likert-scale questionnaire. Descriptive, bivariate, and multivariable regression analyses were used to explore the relationship of demographic, regional, and professional data with predictors of prescribing behaviors.

Results: Our study included 213 emergency physicians, with the majority (62.4%, n=133) aged between 23 and 30 years. While 72.8% (n=155) of participants were male, female physicians represented 27.2% (n=58) of the sample. The analysis revealed that younger physicians, as well as people with one to two years of experience after post-graduation, had a higher opioid prescribing score (OPS). Prior analgesic use, pain severity, and physical examination findings were the most common clinical factors. No significant associations were found for age, gender, practice level, or region. Male physicians showed slightly higher OPS than female physicians (B = 2.53, p = .167, 95%CI -1.07, 6.13). However, the association was not statistically significant. Physicians working more than 16 shifts per month also demonstrated a non-significant trend toward higher OPS compared to those with 12 or fewer shifts (B = 4.91, p = .086, 95%CI -0.71, 10.53). In contrast, fewer years since graduation significantly predicted higher OPS (β = -7.81, p = 0.029) in the multivariable regression. Additionally, gender, practice level, or region revealed no significant differences.

Conclusion: Emergency physicians with fewer years of experience and younger ages are more willing to prescribe opioids mainly directed by clinical causes in Saudi Arabia. These findings call for more focused educational interventions and standardized guidelines in order to promote safe opioid use and reduce potential overprescribing behavior within society.

## Introduction

Among all the different presenting complaints, the most common is pain. Notably, 78% of emergency department (ED) visits are linked to pain [1-2]. While it is not always dangerous, pain can negatively affect many aspects of life, especially mood and daily function. A World Health Organization (WHO) study reported that patients with chronic pain had a four times greater tendency to experience depression or anxiety. Additionally, pain can result in doubling the difficulty of working for most people [3]. While it is important to identify the definite the definitive diagnosis causing pain, pain relief is still considered a priority in many conditions. Alleviation of pain can benefit patients in many ways. For instance, analgesics can reduce pain-related tachycardia in some patients with cardiac conditions, including myocardial infarction and aortic dissection. Moreover, relieving the pain can facilitate patients’ examination in some cases, such as kidney stones and perforated ulcers. Furthermore, higher satisfaction rates of treatment plans are reported after pain relief, which subsequently results in high follow-up adherence rates [4,5]. Subsequently, pain control is considered one of the markers for evaluating healthcare quality in the ED [6].

The choice of modality usually depends on symptom relief, few side effects, and non-interference with other drugs [7]. While many analgesic agents are available, systemic analgesics, including nonsteroidal anti-inflammatory drugs or narcotics, are the primary choice in most emergency cases [8,9]. While opioid pain relievers are considered an acceptable management in the outpatient setting, opioids are also frequently used by physicians in EDs, especially in patients under 40 years [10,11]. The nature of an emergency setting with limited resources and time increases the difficulty of opioid prescribing decisions. For example, many opioids are even prescribed without advancing the established patient-doctor relationships [12]. Remarkably, opioids are administered to one-third of all ED cases, either given in the ED or prescribed at hospital discharge, and increased by 10.2% between 2001 and 2010 [13]. Still, there is a widespread agreement about the insufficient pain relief in most EDs [14]. On the other hand, opioid use disorders have reached epidemic levels in many countries nowadays [15]. The relationship between ED opioid prescribing and opioid use disorders is not well established. Thus, it is vital to consider the adverse effects of opioids, such as chronic opioid use, the risk of opioid ingestion by others, and opioid use disorder [16].

Many factors can influence the opioid prescribing behavior of emergency physicians. These factors include provider evaluation of pain features, patient-based factors, as well as other regulations related to health systems, practice, and policy. Satisfaction scores are also an important factor affecting prescribing behavior. Many physicians attributed high prescribing rates to the fear of getting negative satisfaction scores. Nevertheless, opioid prescribing guidelines and opioid restrictions are taken into consideration by many emergency physicians [17]. Our study aims to investigate the factors that influence emergency physicians’ decision-making when prescribing opioids in Saudi Arabia. Moreover, we aim to assess the impact of emergency physicians' beliefs when it comes to prescribing opioids.

## Materials and methods

This was a cross-sectional study conducted over a one-year period from February 7, 2024, to February 7, 2025. It was initially conducted at the College of Medicine, Taibah University, Medina, Saudi Arabia, and subsequently distributed to additional medical centers across Saudi Arabia. The study was approved by the College of Medicine Research Ethics Committee (CM-REC), Taibah University (study ID TU-24-13, dated February 7, 2024)

Eligibility criteria

Inclusion criteria were practicing emergency physicians within the geographic landmarks of Saudi Arabia, regardless of their position or workplace, whether it was in governmental or private facilities. The exclusion criteria were non-emergency physicians and physicians working outside of Saudi Arabia.

Sample size

Our study targeted emergency physicians in Saudi Arabia. It is estimated that the number of emergency physicians in the country is 448, based on the statistical yearbook of 2021 published by the Ministry of Health, Saudi Arabia [18]. Subsequently, it was estimated that the needed sample size is equal to 208 with a confidence level of 95%, a margin of error equal to 5% using Raosoft software (Raosoft Inc., Seattle, Washington, United States).

A total of 285 individuals voluntarily participated in the survey, all of whom consented to take part. Among them, 213 (74.7%) identified as emergency physicians, while 72 (25.3%) did not. For the purpose of this study, only data from the 213 emergency physicians were included in the final analysis to ensure alignment with the study’s focus on opioid prescribing behaviour in ED settings.

Study tool

An online questionnaire survey form was used based on the published questionnaire in Alrajhi et. al.'s study [17] (see Appendices). The questionnaire consisted of 28 items, of which six were focused on collecting demographic and professional characteristics of the participating emergency physicians, such as age, gender, level of practice, region of work, and years since graduation. These items were excluded from the scoring calculation.

The remaining 22 items were designed to assess physicians’ beliefs, attitudes, and decision-making influences related to opioid prescribing in emergency settings. These items followed a five-point Likert scale format, with response options ranging from "Strongly Disagree" to "Strongly Agree," numerically coded from 1 to 5. All items were framed such that higher scores reflected a stronger agreement or greater influence in the decision-making process.

To compute the opioid prescribing score (OPS), responses to the 22 Likert-scale items were summed, yielding a total score range between 22 and 110. A higher OPS indicated greater susceptibility to various influences or attitudes regarding opioid prescription in emergency medicine. This scoring system enabled the conversion of qualitative attitudes into a structured numerical value, supporting statistical analysis of the patterns and factors associated with prescribing behavior.

The OPS reflects the overall degree to which various clinical, contextual, and psychosocial factors influence an emergency physician's decision to prescribe opioids. A higher OPS indicates greater susceptibility or responsiveness to multiple influences, suggesting that the physician is more likely to be affected by both clinical cues (e.g., pain scores, exam findings) and non-clinical factors (e.g., patient demographics, prescribing culture). In contrast, a lower OPS suggests more selective or restrained influence, indicating that the physician may rely more strictly on clinical guidelines or exhibit greater caution in opioid prescribing decisions. This score helps quantify the variability in prescribing behavior across practitioners.

Statistical analysis

Descriptive Statistics

Descriptive analyses were conducted to summarize participant characteristics and the distribution of key variables. Categorical variables are presented as frequencies and percentages, while continuous variables are described using means and standard deviations (SD) or medians and interquartile ranges (IQR), depending on the normality of the data.

Inferential Statistics

The normality of continuous data was assessed using the Kolmogorov-Smirnov test, which indicated that the data were not normally distributed. As a result, non-parametric tests were employed for group comparisons: the Mann-Whitney U test was used for comparisons between two groups, and the Kruskal-Wallis test was applied for comparisons across more than two groups. Additionally, multiple linear regression analysis was conducted to identify independent demographic and professional predictors of the OPS. A p-value of less than 0.05 was considered statistically significant. All statistical analyses were performed using IBM SPSS Statistics for Windows, version 27.0.1 (IBM Corp., Armonk, New York, United States).

## Results

Demographic and professional characteristics of emergency physicians participating in the study

Among the participating emergency physicians, the majority (62.4%) were aged between 23 and 30 years, followed by 18.3% aged 31-35 years, with progressively fewer participants in older age groups. Male physicians made up a significant portion of the sample at 72.8%, while females represented 27.2%. In terms of workload, more than half of the participants (53.5%) reported working 13-16 shifts per month, with 32.9% working more than 16 shifts, and only 13.6% working 12 or fewer. Regarding experience, 41.8% of the respondents had graduated within the last one to two years, while 26.8% had three to four years of post-graduate experience, and fewer had longer professional histories, with 13.6% having graduated more than 10 years ago. The participants held various roles within emergency medicine: the largest groups included Resident R1 (25.8%) and Staff Physicians/Service Residents (23.5%), with smaller proportions of Board-Certified emergency physicians (10.3%) and Consultants (12.7%). Geographically, the physicians were predominantly practicing in the Central (35.2%) and Western (35.2%) regions, followed by the Eastern (18.8%), Northern (6.1%), and Southern (4.7%) regions. This distribution highlights a young and relatively early-career workforce with diverse levels of training and regional representation (Table 1).

**Table 1:** Demographic and professional characteristics of emergency physicians participating in the study (N = 213) EM: emergency medicine

Characteristics		Frequency	Percentage
Age (years)	23-30	133	62.4%
	31-35	39	18.3%
	36-40	25	11.7%
	41-50	12	5.6%
	51-60	4	1.9%
Gender	Female	58	27.2%
	Male	155	72.8%
Shifts Per Month	≤12 Shifts	29	13.6%
	13-16 Shifts	114	53.5%
	> 16 Shifts	70	32.9%
Years Since Graduation	1-2	89	41.8%
	3-4	57	26.8%
	5-10	38	17.8%
	> 10	29	13.6%
Level of Practice	EM Board-Certified (Assistant, Associate)	22	10.3%
	EM Consultant	27	12.7%
	Resident R1	55	25.8%
	Resident R2	19	8.9%
	Resident R3	19	8.9%
	Resident R4	21	9.9%
	Staff Physicians, Service Resident	50	23.5%
Region of Practice	Central Region	75	35.2%
	Eastern Region	40	18.8%
	Northern Region	13	6.1%
	Southern Region	10	4.7%
	Western Region	75	35.2%

Emergency physicians’ responses to factors influencing opioid prescribing decisions

The table outlines how various factors influence emergency physicians’ opioid prescribing decisions, using a five-point Likert scale ranging from "Strongly Disagree" to "Strongly Agree." Clinical indicators such as the patient’s apparent level of distress (with 69.5% agreeing or strongly agreeing) and vital signs or physical exam findings (63.3% agreement) were among the most strongly endorsed influences. Similarly, the patient’s reported pain score (55.9% agreement) and the type and amount of previously administered medications (69.5% agreement) were also widely recognized as important considerations. Factors like proven substance abuse (59.2% agreement) and opioid history or abuse reputation (58.2% agreement) were also influential for a majority. Conversely, demographic characteristics such as the patient’s age, gender, or nationality received lower agreement, with only 38.1% agreeing or strongly agreeing, and a notable portion (33.8%) expressing disagreement. Concerns about side effects and addiction also played a role, with over 60% of physicians indicating these affected their decisions. Less agreement was seen regarding external influences such as friends’ or family experiences (only 22.1% agreement) and the emergency department prescribing culture (30.1% agreement). Notably, a sizable proportion expressed concern about “doctor shopping” (41.3% agreement) and non-medical use (50.7% agreement). Confidence in identifying addiction (55.4% agreement) and “doctor shopping” (37.6% agreement) varied, reflecting possible uncertainty in clinical judgment. Overall, the findings suggest that clinical presentation and substance use history are the most influential factors in opioid prescribing decisions, while external pressures and demographic variables are less commonly considered (Table 2).

**Table 2:** Emergency physicians’ responses to factors influencing opioid prescribing decisions

Factors Influencing Opioid Prescription and Dosing	Strongly Disagree		Disagree		Neutral		Agree		Strongly Agree	
	Frequency	Percentage	Frequency	Percentage	Frequency	Percentage	Frequency	Percentage	Frequency	Percentage
Diagnosis Thought to Be the Cause of Pain	16	7.5%	29	13.6%	57	26.8%	61	28.6%	50	23.5%
Patient's Reported Pain Score	5	2.3%	24	11.3%	65	30.5%	75	35.2%	44	20.7%
Vital Signs & Physical Exam Findings	8	3.8%	23	10.8%	47	22.1%	61	28.6%	74	34.7%
Patient’s Apparent Level of Distress	4	1.9%	20	9.4%	41	19.2%	73	34.3%	75	35.2%
Patient’s Age, Gender, or Nationality	39	18.3%	33	15.5%	60	28.2%	54	25.4%	27	12.7%
Lab or Imaging Results	29	13.6%	34	16.0%	50	23.5%	48	22.5%	52	24.4%
Opioid History & Abuse Reputation	16	7.5%	21	9.9%	52	24.4%	76	35.7%	48	22.5%
Proven Substance Abuse	16	7.5%	20	9.4%	51	23.9%	66	31.0%	60	28.2%
Other Current Medications	8	3.8%	31	14.6%	60	28.2%	62	29.1%	52	24.4%
Specific Request for Opioids	33	15.5%	27	12.7%	52	24.4%	54	25.4%	47	22.1%
Overall Patient Satisfaction	19	8.9%	31	14.6%	57	26.8%	63	29.6%	43	20.2%
Type and Amount of Previous Pain Control Medications Given	8	3.8%	17	8.0%	40	18.8%	80	37.6%	68	31.9%
Concern About Side Effects	10	4.7%	24	11.3%	50	23.5%	64	30.0%	65	30.5%
Concern About Promoting Addiction	14	6.6%	38	17.8%	67	31.5%	50	23.5%	44	20.7%
Concern About “Doctor Shopping”	29	13.6%	41	19.2%	55	25.8%	46	21.6%	42	19.7%
Concern About Non-Medical Use	28	13.1%	19	8.9%	58	27.2%	56	26.3%	52	24.4%
Belief that ED Providers Are Source of Misuse	45	21.1%	52	24.4%	58	27.2%	37	17.4%	21	9.9%
Confidence Identifying “Doctor Shopping”	17	8.0%	44	20.7%	72	33.8%	57	26.8%	23	10.8%
Confidence Identifying Addiction to Opioids	14	6.6%	21	9.9%	60	28.2%	77	36.2%	41	19.2%
Preference to Over-Prescribe Rather than Under-Treat Patients	45	21.1%	35	16.4%	63	29.6%	51	23.9%	19	8.9%
Influence of Friends/Family Experience	72	33.8%	39	18.3%	55	25.8%	36	16.9%	11	5.2%
Influence of ED Prescribing Culture and Colleagues	36	16.9%	40	18.8%	73	34.3%	50	23.5%	14	6.6%

**Figure 1:**
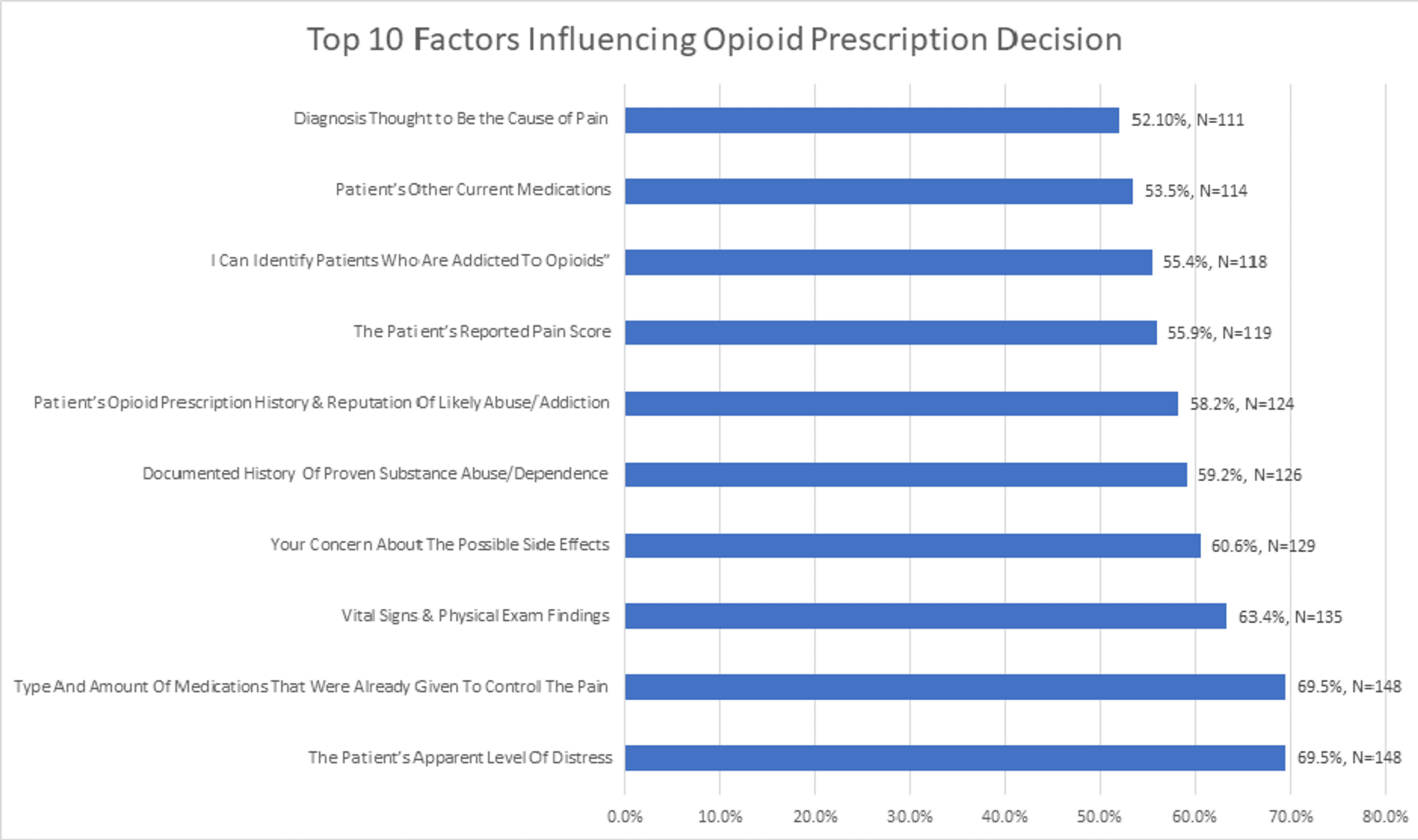
Percentage of emergency physicians who agreed or strongly agreed that each factor influences their decision to prescribe opioids.

Figure 1 displays the top 10 most influential factors, based on combined agreement (Agree + Strongly Agree) percentages. Clinical indicators such as the patient’s level of distress (69.5%) and previously administered medications (69.5%) were the most endorsed. Other prominent factors included physical exam findings (63.3%), concerns about side effects or addiction (>60%), and substance use history (59.2-58.2%). Demographic factors and external influences received much lower agreement and are not shown here.

OPS by demographic and professional characteristics of emergency physicians

The OPS, which ranges from 22 to 110, was used to evaluate how strongly various clinical, contextual, and psychosocial factors influence emergency physicians' opioid prescribing decisions. Higher scores reflect a greater degree of influence in prescribing behavior, while lower scores suggest a more cautious or guideline-driven approach.

When analyzing OPS across age groups, physicians aged 23-30 years had the highest average score (73.86 ± 11.62), followed closely by those aged 31-35 years (73.21 ± 11.55). In contrast, the lowest average OPS was observed among physicians aged 36-40 years (68.52 ± 8.09). Although this pattern indicates that younger physicians may be more susceptible to a range of prescribing influences, the difference was not statistically significant (p = 0.128). In terms of gender, male physicians had a slightly higher average OPS (73.92 ± 11.12) compared to female physicians (71.67 ± 13.02); however, this difference was not statistically significant (p = 0.074), indicating no meaningful difference in opioid prescribing influence between genders.

Workload, as measured by the number of shifts per month, showed that physicians working more than 16 shifts had a higher average OPS (75.96 ± 12.23) compared to those working 12 or fewer shifts (70.31 ± 14.94); however, this difference was not statistically significant (p = 0.071), indicating no conclusive association between monthly shift load and opioid prescribing influence in this sample.

A statistically significant difference was observed with regard to years since graduation (p = 0.030). Physicians who had graduated within the past one to two years had the highest average OPS (75.07 ± 11.07), followed by those with three to four years of experience (73.26 ± 12.06). In contrast, those with 5-10 years of experience had the lowest OPS (69.26 ± 10.61). This suggests that more recent graduates may be more impressionable or responsive to multiple influences during clinical decision-making, potentially due to their limited experience or heightened exposure to recent training on opioid stewardship.

When stratified by level of practice, OPS values varied across groups, though the differences were not statistically significant (p = 0.525). Resident physicians, particularly those in their second year (R2), had the highest average score (75.58 ± 10.77), followed by staff physicians and service residents (75.40 ± 12.81). Meanwhile, EM board-certified physicians reported lower scores (72.55 ± 10.55), and Resident R3 physicians showed the lowest among all categories (69.42 ± 11.45). These results may reflect variations in clinical autonomy, confidence, or institutional oversight across different levels of training.

Finally, regional differences were explored based on the physicians' location of practice. While the differences were not statistically significant (p = 0.149), physicians practicing in the Southern (76.70 ± 12.04) and Western (75.44 ± 12.06) regions had higher OPS compared to those in the Northern region (68.62 ± 9.03). These regional disparities could be related to differing prescribing cultures, institutional policies, or patient demographics.

Overall, while several demographic and professional factors showed observable trends in OPS, only the number of years since graduation reached statistical significance. This finding underscores the potential impact of clinical experience and medical training on opioid prescribing behavior. The relatively uniform distribution of OPS across other variables highlights the need for targeted education and system-level interventions to promote safe and consistent prescribing practices across all physician groups (Table 3).

**Table 3:** Opioid prescribing score (OPS) by demographic and professional characteristics of emergency physicians U: Independent Samples Mann-Whitney U test; K: Independent Samples Kruskal-Wallis test; *p<0.05, significant

Characteristics		Opioid Prescribing Score (OPS) (22-110)					
		Mean	SD	Median	IQR	P value^U^	P value^K^
Age (years)	23-30	73.86	11.62	75.00	66.00-81.00		0.128
	31-35	73.21	11.55	73.00	67.00-84.00		
	36-40	68.52	8.09	67.00	64.00-72.00		
	41-50	76.75	17.63	70.00	63.50-90.50		
	51-60	75.50	9.75	73.50	69.00-82.00		
Gender	Female	71.67	13.02	70.00	63.00-77.00	0.074	
	Male	73.92	11.12	74.00	67.00-81.00		
Shifts Per Month	≤ 12 Shifts	70.31	14.94	66.00	62.00-77.00		0.071
	13-16 Shifts	72.44	10.10	73.00	66.00-77.00		
	> 16 Shifts	75.96	12.23	75.00	66.00-85.00		
Years Since Graduation	1-2	75.07	11.07	76.00	69.00-82.00		0.030*
	3-4	73.26	12.06	74.00	65.00-82.00		
	5-10	69.26	10.61	69.00	64.00-75.00		
	> 10	73.28	13.29	70.00	65.00-75.00		
Level of Practice	EM Board-Certified (Assistant, Associate)	72.55	10.55	69.00	64.00-77.00		0.525
	EM Consultant	72.96	15.71	72.00	62.00-81.00		
	Resident R1	73.42	10.10	75.00	69.00-79.00		
	Resident R2	75.58	10.77	75.00	66.00-82.00		
	Resident R3	69.42	11.45	70.00	63.00-79.00		
	Resident R4	70.71	8.42	72.00	67.00-76.00		
	Staff Physicians, Service Resident	75.40	12.81	72.50	65.00-86.00		
Region of Practice	Central Region	72.49	11.64	73.00	64.00-80.00		0.149
	Eastern Region	71.50	11.25	70.00	64.00-79.00		
	Northern Region	68.62	9.03	69.00	65.00-74.00		
	Southern Region	76.70	12.04	74.00	66.00-88.00		
	Western Region	75.44	12.06	75.00	68.00-82.00		

Multivariable linear regression analysis of factors associated with OPS

A multivariable linear regression analysis was conducted to identify demographic and professional factors associated with emergency physicians’ OPS. The model included age, gender, number of shifts per month, years since graduation, level of practice, and region of practice. Higher OPS values indicate greater susceptibility to prescribing influences, whereas lower scores reflect more restrained or guideline-adherent prescribing behaviors.

The only statistically significant predictor of OPS was years since graduation. Specifically, physicians with 5-10 years of experience post-graduation had significantly lower OPS values compared to those with one to two years of experience (B = -7.81, p = .029, 95%CI -14.81, -0.80). Specifically, physicians with 5-10 years of experience post-graduation had significantly lower OPS values compared to those with one to two years of experience (B = -7.81, p = .029, 95%CI -14.81, -0.80). This suggests that mid-career emergency physicians may be less influenced by external or subjective factors in opioid prescribing, potentially reflecting greater clinical confidence, experience-based decision-making, or habituation to practice norms. In contrast, early-career emergency medicine residents, particularly those with one to two years since graduation, may be more prone to prescribing opioids or more easily influenced by situational, emotional, or non-clinical factors during prescribing decisions. Other experience groups, including those with more than 10 years since graduation, did not demonstrate significant differences.

No significant associations were found for age, gender, practice level, or region. Although not statistically significant, male physicians showed slightly higher OPS than female physicians (B = 2.53, p = .167, 95%CI -1.07, 6.13). Physicians working more than 16 shifts per month also demonstrated a non-significant trend toward higher OPS compared to those with 12 or fewer shifts (B = 4.91, p = .086, 95%CI -0.71, 10.53) (Table 4).

**Table 4:** Multivariable linear regression analysis of factors associated with opioid prescribing score. Dependent variable: opioid prescribing score (OPS) (22-110); *p<0.05, significant

Characteristics		Unstandardized Coefficients		Standardized Coefficients	t	95.0% CI for B		P value
		B	Std. Error	Beta		Lower	Upper	
Age (years)	23-30	Ref	Ref	Ref	Ref	Ref	Ref	Ref
	31-35	2.624	3.011	.087	.872	-3.315	8.564	0.385
	36-40	-2.450	4.051	-.068	-.605	-10.440	5.541	0.546
	41-50	4.587	6.130	.091	.748	-7.505	16.678	0.455
	51-60	1.222	7.775	.014	.157	-14.113	16.556	0.875
Gender	Female	Ref	Ref	Ref	Ref	Ref	Ref	Ref
	Male	2.529	1.824	.097	1.387	-1.069	6.126	0.167
Shifts Per Month	≤ 12 Shifts	Ref	Ref	Ref	Ref	Ref	Ref	Ref
	13 - 16 Shifts	2.098	2.651	.090	.791	-3.131	7.327	0.430
	> 16 Shifts	4.910	2.850	.198	1.723	-.711	10.531	0.086
Years Since Graduation	1-2	Ref	Ref	Ref	Ref	Ref	Ref	Ref
	3-4	-.972	2.510	-.037	-.387	-5.923	3.978	0.699
	5-10	-7.806	3.551	-.256	-2.198	-14.810	-.801	0.029*
	> 10	-5.098	5.329	-.150	-.957	-15.609	5.412	0.340
Level of Practice	EM Board-Certified (Assistant, Associate)	Ref	Ref	Ref	Ref	Ref	Ref	Ref
	EM Consultant	-1.085	3.710	-.031	-.292	-8.403	6.234	0.770
	Resident R1	-4.501	3.772	-.169	-1.193	-11.940	2.938	0.234
	Resident R2	-2.585	4.256	-.063	-.607	-10.980	5.810	0.544
	Resident R3	-6.582	4.249	-.161	-1.549	-14.964	1.800	0.123
	Resident R4	-4.049	3.905	-.104	-1.037	-11.752	3.654	0.301
	Staff Physicians, Service Resident	-1.353	3.517	-.049	-.385	-8.290	5.583	0.701
Region of Practice	Central Region	Ref	Ref	Ref	Ref	Ref	Ref	Ref
	Eastern Region	-2.214	2.469	-.074	-.897	-7.085	2.656	0.371
	Northern Region	-5.056	3.655	-.104	-1.383	-12.265	2.154	0.168
	Southern Region	2.416	4.046	.044	.597	-5.564	10.397	0.551
	Western Region	2.374	2.006	.097	1.184	-1.583	6.332	0.238

Overall, these findings suggest that while most demographic and practice-related factors do not significantly impact opioid prescribing attitudes of emergency physicians, years since graduation of the prescribing physician may play a meaningful role.

## Discussion

Our study of 213 Saudi emergency physicians revealed that younger and less experienced Saudi emergency physicians had higher OPS, reflecting a more liberal approach to opioid use. Their prescribing decisions were mainly influenced by clinical cues such as pain scores, patient distress, and prior analgesic use, while demographic factors and ED culture had little impact. Years since graduation showed a strong inverse relationship with OPS, indicating that more experienced physicians were more conservative, likely due to greater clinical caution and awareness of opioid risks. This pattern suggests a generational shift in prescribing attitudes, with junior physicians more inclined toward opioid use and senior physicians favoring restraint. This observation resonates with prior work in other contexts; For example, Alrajhi et al. (2023) also reported that physician age and years of practice significantly affected opioid prescribing behavior among Saudi emergency physicians [17].

Similarly, the strong influence of patient distress and pain severity on prescribing observed in our study mirrors findings that clinicians frequently cite objective pain cues as primary determinants. Alrajhi et al. found that “the highest self-rated prescribing factors were patients’ distress level and the previously given medications” [17]. In our sample, high pain scores, visible suffering, and prior analgesic use likewise drove decisions; emergency physicians appeared to base opioid use on clinical need cues rather than external pressures. Other studies of ED clinicians have similarly highlighted the primacy of clinical assessment. For example, Hoppe and colleagues found that even after adjusting for patient characteristics, large variation in opioid prescribing persisted, indicating that individual clinician judgement (often informed by patient symptoms and history) is a major driver [19]. In short, our results reinforce that Saudi emergency physicians give the greatest weight to in-the-moment clinical indicators (pain intensity and distress) when deciding on opioids, and far less weight to patient demographics or unit culture.

The relative lack of influence of patient demographics in our study is noteworthy. In many Western settings, socio-demographic factors markedly affect analgesic care. For instance, United States data document racial and ethnic disparities in pain management: Black or Hispanic patients are significantly less likely than White patients to receive opioid analgesia [20]. In contrast, our analysis suggested that Saudi physicians’ OPS did not differ substantially by patient age or gender (nor by ED location or patient load). This could reflect cultural and systemic differences, as the Saudi healthcare environment and patient population may be more homogeneous, or physicians may follow more uniform protocols for pain. It may also indicate that, in our context, overt demographic biases play a smaller role than in diverse Western ED populations.

Our finding that departmental or regional culture exerted little effect contrasts with some reports that institutional norms or policies can shape prescribing. For example, surveys in other countries have found that local guidelines or peer practice patterns influence clinicians’ opioid use. In Dutch EDs, Kraaijvanger et al. noted wide variation and inconsistent guideline use across hospitals [21]. However, in our data, neither region of practice (central vs. western Saudi Arabia) nor self-reported workplace culture had a strong impact on OPS. This suggests that individual physician attitudes and the immediate clinical scenario override broader workplace norms. It is possible that Saudi EDs lack well-established local opioid policies, so individual judgment prevails, or that any existing protocols are applied uniformly across the country.

Globally, opioid prescribing in the ED is shaped by the interplay of evidence-based guidelines and cultural context. In the United States and Canada, the opioid epidemic has led to focused efforts to cut back on opioid use. United States guidelines (e.g., CDC 2022) now highlight maximizing non-opioid therapies and using opioids only when benefits clearly outweigh risks [22]. Accordingly, many studies in the United States report declining ED opioid prescriptions over the last decade. For example, Gleber et al. observed a steady drop in ED opioid prescriptions from 37.8% to 13.3% (2012-2018) with a concurrent rise in non-opioids [23]. This trend reflects heightened physician caution; indeed, United States emergency medicine societies advise using opioids only for severe, refractory pain [11]. Our finding that older physicians, who trained before the current anti-opioid climate, prescribe less may parallel the United States pattern that more seasoned clinicians have seen the negative consequences and thus practice conservatively. On the other hand, younger United States physicians who trained more recently have also been admonished about the opioid crisis, which might make them more reluctant. In our Saudi sample, however, the opposite appeared true, suggesting local differences in training emphasis.

In Canada, pediatric ED data indicate that clinicians remain willing to use opioids for short-term acute pain despite the opioid crisis, with physicians reporting minimal concern about dependence in this context [24]. In our Saudi cohort, higher OPS among recent graduates suggests comparatively greater readiness to consider opioids for acute pain when clinically indicated, whereas more experienced physicians appeared more conservative. Our observations highlight that emergency physicians in different health systems may vary in their thresholds for prescribing opioids, but concerns about the opioid crisis do not necessarily preclude the use of short-term opioids for acute pain.

European data are sparser but instructive. **In many European countries, opioid pr**escribing has historically been much more restrained than in North America, though some nations (e.g., the United Kingdom, the Netherlands) have seen increasing use [25]. A recent Dutch survey found that few Dutch EDs fully adhered to stringent opioid guidelines: only 58% had any local protocols, and most did not follow the national standards closely [21]. This is echoed in our findings, in that Saudi ED prescribing remains cautious relative to Western usage as Middle Eastern availability of opioids is generally low [26], and clinicians may be inherently more conservative. However, Saudi practice also appears to be changing, as evidenced by our relatively high OPS scores among juniors. Whether this trend mirrors any European shift is unclear. It may be that, like Dutch EDs, Saudi EDs are beginning to use more opioids, especially when pain is severe, even without strict guideline implementation. As in Europe, individual clinician judgment seems to drive decisions more than formal policy in Saudi EDs.

Within the Arabian Gulf and neighboring regions, opioid use for acute pain is typically lower than in the West, due to regulatory controls and cultural attitudes. The WHO/Global Opioid Policy Initiative reports that, except for Israel, opioid availability is “low throughout most of the Middle East” [26]. In Saudi Arabia and nearby countries (United Arab Emirates (UAE), Qatar, Jordan), opioids are tightly regulated and often stigmatized, which can limit both prescribing and patient expectations [27]. Our study’s finding that ED clinicians focus on clinical cues as pain severity and distress is consistent with a setting where opioids are reserved for clear medical need rather than routine use.

Data from other Gulf countries is limited. In the UAE and Qatar, small studies indicate that fear of addiction and legal consequences contributes to under-treatment of pain [28,29]. Regional surveys, including those from Saudi Arabia, similarly report inadequate opioid knowledge and reluctance to prescribe strong opioids [30,31]. Our finding that more experienced or educated physicians tend to prescribe fewer opioids (lower OPS) aligns with evidence that better-trained providers use opioids more judiciously [32]. In contrast, higher OPS among junior Saudi EPs may reflect recent training emphasizing patient-centered pain management [33].

Cultural factors likely also play a role. In Saudi Arabia, where social and religious norms strongly discourage the use of intoxicating substances, non-medical drug use is illegal, and addiction carries considerable stigma, both patients and clinicians may have firm preconceptions about narcotics and a general tendency toward cautious use. The fact that patient demographics did not predict OPS suggests that Saudi physicians may not differentiate treatment based on such social factors, possibly due to cultural norms of equity or to a lack of conscious bias. However, broader culture, such as public attitudes toward addiction or regulatory oversight, may still shape prescribing philosophies. For example, widespread recommendations on avoiding addiction may make any ED opioid prescribing feel exceptional, reinforcing reliance on objective pain evidence. Thus, while our EPs seemed guided by clinical cues, we cannot separate that from the background where opioids are treated with extra caution.

Implications for policy and practice

Our findings have multiple practical implications. The association of higher OPS with younger physicians suggests that medical education and ongoing training are critical key drivers. If emerging physicians are more liberal in opioid use, targeted education about risk stratification, non-opioid alternatives, and safe prescribing may be needed to ensure balance. Integration of Saudi or regional guidelines into undergraduate and residency curricula could standardize practice. Highlighting pain as a complex biopsychosocial phenomenon may also help junior doctors consider nonpharmacologic therapies and multimodal analgesia before opioids, in line with CDC advice [22].

The lack of influence from patient demographics and ED culture suggests that interventions to change prescribing should focus on individual decision-making. For instance, implementing clinical decision support (such as pain treatment algorithms that include non-opioid pathways) could leverage the strong role of clinical cues we observed. Conversely, because emergency physicians in our setting rely on pain scores and distress, enhancing the accuracy of pain assessment, through training or standardized tools, could improve patient care. That physicians heavily weigh prior analgesic attempts implies that better documentation of prior medications using prescription drug monitoring programs might further refine choices.

In addition, this topic is particularly important for Saudi Arabia at this stage of its health system development. The country is rapidly expanding emergency and trauma services, pain clinics, and oncology and surgical programs, which will inevitably increase exposure to opioids for both acute and chronic pain. At the same time, there are growing concerns among clinicians about under-treated pain on one side and the potential for emerging misuse, diversion, or dependence on the other, especially in a young population with high rates of road-traffic injuries and musculoskeletal pain. Mapping current attitudes in EDs, therefore, offers an opportunity to intervene early, through tailored guidelines, education, and monitoring, before prescribing patterns become entrenched, and to design policies that avoid simply importing North American or European responses that may not fit the Saudi cultural and regulatory context.

Finally, policymakers should note that regional and local contexts matter. Saudi Arabia has the opportunity to craft balanced opioid policies that reflect the needs of its population. Our data suggest that Saudi emergency physicians are already cautious, especially older doctors, but younger clinicians may benefit from guidance on when opioids are truly indicated. Health authorities might consider national consensus guidelines for ED pain management, incorporating WHO/CDC principles, to reduce unwarranted variation. In healthcare systems where cultural aversion to opioids is strong, public education on the safe use of pain medication may also be warranted to align patient expectations with best practice. Importantly, any policy should ensure access; while caution is necessary, unnecessary under-treatment of pain is also a risk. Careful monitoring of prescribing trends in Saudi EDs, as well as follow-up studies on patient outcomes, would help conclude whether changes in physician attitudes translate to optimal pain relief and minimal harm.

Limitations

This study has several limitations that warrant consideration. First, it relied on a self-administered survey, which is inherently subject to response bias and social desirability bias; physicians may have over- or under-reported their true prescribing behaviors or attitudes. Second, while the OPS provided a structured measure of attitudes, it reflects self-reported influences rather than actual prescribing practices and thus cannot fully capture real-world clinical decision-making. Third, the cross-sectional design precludes causal inference, limiting the ability to determine whether specific demographic or professional characteristics directly influence prescribing behavior. Fourth, the sample size was modest and drawn from a finite pool of emergency physicians, which is itself limited in Saudi Arabia; as a result, the precision of some estimates is restricted, and replication in larger, multi-center cohorts is needed.

Additionally, participants were primarily younger and early-career emergency physicians, which may limit the generalizability of findings to more experienced physicians or other specialties. Moreover, the sample was drawn from physicians practicing in a localized region in Saudi Arabia; cultural norms, regulatory frameworks, and healthcare system characteristics may differ from those in other regions within the Kingdom, neighboring countries, or globally, further limiting external comparability. Finally, certain potentially relevant variables, such as institutional opioid prescribing policies, patient case mix, or availability of non-opioid alternatives, were not captured in the survey, which may have provided further insight into the contextual determinants of opioid prescribing.

## Conclusions

This study sheds light on self-reported opioid prescribing patterns among Saudi emergency physicians. Within our sample, younger and less experienced clinicians reported a greater propensity to prescribe opioids and were more likely to be guided primarily by clinical pain indicators, whereas older clinicians reported more conservative prescribing. These findings describe patterns observed in the surveyed Saudi emergency physicians and should not be extrapolated beyond this context. They may nonetheless help inform Saudi medical leaders when tailoring local education, guidelines, and policies to support safe and effective pain management in emergency departments.

## Appendices

Study questionnaire

Source: Alrajhi et al., 2023 [17]

1.    Age

·         23-30

·         31-35

·         36-40

·         41-50

·         51-60

·         61-70

2.    Gender

·         Male

·         Female

3.    Number Of Shifts Per Month

·         12 Or Less Shifts

·         13-16 Shifts

·         >16 Shifts

4.    Years Of Practice Since Graduation From Medical School

·         1-2 Years

·         3-4 Years

·         5-10 Years

·         >10 Years

5.    Your Level Of Practice

·         EM Consultant

·         EM Board- Certified (Assistant, Associate)

·         Staff Physicians, Service Residents

·         Resident R4

·         Resident R3

·         Resident R2

·         Resident R1

6.    You Are Currently Practicing In

·         Western Region

·         Central Region

·         Eastern Region

·         Southern Region

·         Northern Region

Which Factors Affect Your Selection Of An Opioid Or Its Dosing (In ED Or Upon Discharge)

(Strongly Disagree = 1, Disagrees = 2, Normal = 3, Agree = 4, Strongly Agree = 5)

7.    Diagnosis thought to be the cause of pain

a.  Strongly Disagree

b.  Disagree

c.  Neutral

d.  Agree

e.  Strongly Agree

8. The patient’s reported pain score

a.  Strongly Disagree

b.  Disagree

c.  Neutral

d.  Agree

e. Strongly Agree

9. Vital signs and physical exam findings

a.  Strongly Disagree

b.  Disagree

c. Neutral

d. Agree

e. Strongly Agree

10. The patient’s apparent level of distress

a. Strongly Disagree

b. Disagree

c.  Neutral

d. Agree

e. Strongly Agree

11.  The patient’s age, gender, or nationality

a. Strongly Disagree

b. Disagree

c. Neutral

d. Agree

e. Strongly Agree

12. Laboratory or imaging results

a. Strongly Disagree

b. Disagree

c. Neutral

d. Agree

e. Strongly Agree

13. Patient’s opioid prescription history and reputation of likely abuse/addiction

a.  Strongly Disagree

b. Disagree

c. Neutral

d. Agree

e. Strongly Agree

14. Documented history of proven substance abuse/dependence

a. Strongly Disagree

b. Disagree

c. Neutral

d. Agree

e. Strongly Agree

15. Patient’s other current medications

a. Strongly Disagree

b. Disagree

c. Neutral

d. Agree

e. Strongly Agree

16. Patient’s specific request for opioids

a. Strongly Disagree

b. Disagree

c. Neutral

d. Agree

e. Strongly Agree

17. Patient’s overall satisfaction

a. Strongly Disagree

b. Disagree

c. Neutral

d. Agree

e. Strongly Agree

18. Type and amount of medications that were already given to control the pain

a. Strongly Disagree

b. Disagree

c. Neutral

d. Agree

e. Strongly Agree

19. Your concern about the possible side effects

a. Strongly Disagree

b. Disagree

c. Neutral

d. Agree

e. Strongly Agree

20.  Your concern about promoting addiction

a. Strongly Disagree

b. Disagree

c. Neutral

d. Agree

e. Strongly Agree

21. Your concern about if the patient is "Doctor shopping"

a. Strongly Disagree

b. Disagree

c. Neutral

d. Agree

e. Strongly Agree

22. Your concern about non-medical use of the medications (addiction, illegal uses)

a. Strongly Disagree

b. Disagree

c. Neutral

d. Agree

e. Strongly Agree

23.  ED providers are possibly a “significant source of opioid medications that are used illegally”

a. Strongly Disagree

b. Disagree

c. Neutral

d. Agree

e. Strongly Agree

24.  I can accurately identify patients who “are doctor shopping”

a. Strongly Disagree

b. Disagree

c. Neutral

d. Agree

e. Strongly Agree

25.  I can identify patients who are addicted to opioids”

a. Strongly Disagree

b. Disagree

c. Neutral

d. Agree

e. Strongly Agree

26.  I would rather over-prescribe opioids and risk illegal use, than under-treat patients’ pain

a. Strongly Disagree

b. Disagree

c. Neutral

d. Agree

e. Strongly Agree

27.  My prescribing behavior of opioids is affected by my friends' and family's experience with these medications

a. Strongly Disagree

b. Disagree

c. Neutral

d. Agree

e. Strongly Agree

28.  My prescribing behavior of opioid is affected by the prescribing culture of my emergency department and colleagues

a. Strongly Disagree

b. Disagree

c. Neutral

d. Agree

e. Strongly Agree
